# The complexities of simple technologies: re-imagining the role of rapid diagnostic tests in malaria control efforts

**DOI:** 10.1186/s12936-016-1083-2

**Published:** 2016-02-05

**Authors:** Uli Beisel, René Umlauf, Eleanor Hutchinson, Clare I. R. Chandler

**Affiliations:** Department of Anthropology, Bayreuth University, Universitätsstraße 30, 95440 Bayreuth, Germany; Department of Sociology, Bayreuth University, Universitätsstraße 30, 95440 Bayreuth, Germany; Department of Global Health and Development, London School of Hygiene & Tropical Medicine, 15-17 Tavistock Place, London, WC1H 9SH UK

**Keywords:** RDTs, Simplicity, Mobility, Standardization, Health technologies, Health infrastructures

## Abstract

**Background:**

Malaria rapid diagnostic tests (RDTs) are assumed to be simple-to-use and mobile technologies that have the capacity to standardize parasitological diagnosis for malaria across a variety of clinical settings. In order to evaluate these tests, it is important to consider how such assumptions play out in practice, in everyday settings of clinics, health centres, drug stores and for community health volunteers.

**Methods:**

This paper draws on qualitative research on RDTs conducted over the last nine years. In particular the study reports on four qualitative case studies on the use of RDTs from Uganda, Tanzania and Sierra Leone, including qualitative interviews, focus group discussions and participant observation.

**Results:**

Results suggest that while RDTs may be simple to use as stand-alone technological tools, it is not trivial to make them work effectively in a variety of economically pressured health care settings. The studies show that to perform RDTs effectively might very well need exactly the infrastructure they were designed to substitute: the medical expertise, organizational capacity and diagnostic and treatment options of well-funded and functioning health systems.

**Conclusions:**

These results underline that successful malaria diagnosis and treatment requires as much investment in general health infrastructure as it does in new technologies.

## Background

This paper examines the implementation and use of mobile malaria rapid diagnostic tests (RDTs) in sub-Saharan Africa. The study draws on empirical material from qualitative studies in Uganda, Tanzania and Sierra Leone. Strong arguments for the introduction of these mobile diagnostic devices have been made within recent public health discourse [1–3]. Firstly, adequate diagnosis is hoped to minimize the long-persisting practice of fever-based management of malaria (‘fever equals malaria’). Such ‘blanket’ management of fevers is argued to increase the risk of leaving non-malarial diseases untreated that also have fever as a primary symptom. The phenomenon of concordant symptoms can become a life threatening issue, for example for children afflicted by pneumonia. Secondly, confirmation of malaria with RDTs is hoped to address uncontrolled anti-malarial prescription practices and usage patterns. This promises to avoid drug wastage and thereby reduce a strain on public-health budgets, as well as reduce pressure for drug resistance. Thirdly, the mobility of diagnostic means is important, as research into local treatment practices has revealed the majority of all suspected malaria cases continue to be treated outside formal/public health care sector.

The gold-standard of malaria diagnosis for clinical care remains microscopic diagnosis [4]. However, due to shortages of laboratories, electricity and specialized lab personnel, microscopic diagnosis is not realistically attainable for many settings in developing countries, in particular rural and infrastructurally weak areas. Thus, RDTs are intended to complement (but also, as will been shown, in some cases replace) microscopic services, to make parasite-based diagnosis a standard operating procedure at all points of care [5]. RDTs are considered able to deliver the above promises mainly due to two features: their mobility and simplicity. Due to their size and low requirements for supportive technology (RDTs do not need electricity or cooling), RDTs can travel to remote areas where no laboratory services have been available. They are deemed to be easy to perform in comparison to microscopy or other diagnostic technology: a drop of blood is inserted in a hole on the test, a few drops of buffer in a second hole and 15 or so minutes later the results can be read. Similar to pregnancy tests, the binary code the tests operate with is expressed in a combination with control lines that determine either a positive or negative result. A positive test result provides evidence to access malaria treatment. The alternative scenario in which the patient/client is presented with a negative test result is intended to trigger an alternative pathway of care: identifying and treating alternative causes clinically, sending for further diagnostics, and/or referral to a higher level health facility in order to determine and manage the cause of symptoms.

The tests’ mobility and simplicity are regarded as particularly compelling features for their use outside of formal health facilities. RDTs are desirable when they can be performed with limited training, can be relatively independent of supporting infrastructure, and can accommodate high patient loads, staff shortages and suboptimal infrastructure [6]. Thus, it is argued that RDTs can be successfully introduced along pre-given institutional divisions and organizational structures in health systems, with a promise to standardize parasite-based diagnosis inside and outside laboratories. However, the advent of RDTs adds an experimental dimension to patterns of pre-existing diagnostic capacities and practices.

Much of the early debate on introduction of RDTs was concerned with the varying performance of RDT products. Discussions focused on the quality of product manufacturing, but also on how high temperature changes during transport and storage can potentially affect the sensitivity and specificity of the technology in detecting malaria antigens. Another critical field of discussion was the actual performance of tests in practice and how health workers would be able to manage RDTs within existing clinical structures and work routines. In their attempt to trace the evolution of RDTs Frost and Reich state that, “these problems with product performance created considerable uncertainty among local, national, and global actors about whether and where RDTs should be used in health systems in developing countries. For example, when is it appropriate to use RDTs instead of improving microscopy in a particular setting? In what situations are RDTs cost-effective? Should RDTs be used in both the public and private sectors?” [7]. While the questions mainly reflect early stage concerns of introducing a novel technology the empirical examples show that the widespread use of RDTs has not necessarily settled the controversies around these issues. Quite the contrary, as RDTs are used on very different levels in the health system, including community work, lower-level and higher-level public health facilities, as well as increasingly in the private sector, a variety of new and place-specific issues have come up that are widely unanswered, and/or remain overlooked.

This article reflects on users’ attempts to make RDTs work in practice. In the four empirical case studies the focus is on the work that is involved to accommodate and adapt the technology to the everyday routines in the health facilities, drug stores and for community health volunteers. RDTs emerge as a technology demanding considerable attention on an on-going basis, rather than a finished product that simply needs distribution and brief training. The results show how RDTs and the trials around them *have* been transformative, but not in the ways in which the policy debate would suggest. Indeed, the extensive amount of work that are required to render RDTs effective, enables us to question assumptions about the technology’s simplicity, mobility and, in some cases, its suitability. This does not mean that the technology itself has a problem or is insufficient, but rather draws attention to what social studies of technology have termed the co-construction of technology and society [8, 9]. Co-construction means that technological artifacts are shaped by the societies that design, produce and use them. Vice versa the society too is shaped by the technology: “users and technology are seen as two-sides of the same problem” [10]. Seeing technology and users as co-constructed implies a shift from users as passive recipients to active participants. The case studies align with this perspective of co-construction and thus underline the effects that the introduction of a new technology has on its users, as well as the effects of the users and health infrastructures on the functioning of the technology.

The case studies suggest that broad implementation of RDTs across Africa has had many notable effects, but they cannot be shown to be simple to use for a great variety of users. For instance, RDTs provide a significant organizational and time challenge for overstretched health clinic routines in Uganda (case 1). Secondly, while RDTs no doubt have extended the reach of parasitological diagnosis for malaria, standardization has not been uniform either. It is rather a complex negotiation process that depends on the skills and resources of the care personnel, and requires delicate moral negotiation of health care staff between treatment guidelines and the suffering of patients (case 2). Thirdly, while RDTs are no doubt a simple technology in itself, this does not mean they are simple to use for everybody, as this study shows uneducated and unskilled health volunteers in Sierra Leone struggle with correct and safe application of tests (case 3). Further, the use of RDTs in pharmacies can create an aura of medical expertise and legitimacy for pharmacy personnel that is seductive for the business owners as it promises more revenue, but from a medical lens leads to more diagnostic and treatment uncertainties and overuse of medicines, in particular for patients that test RDT negative (case 4). Taken together, the results draw attention to the co-construction of technology and its users. RDTs are not in themselves a simple or fast technology. Its ease and speed is rather defined in practice, when users and the technology interact. Furthermore, RDTs in themselves do not simply introduce standardization or successful parasitological diagnosis.

Standardization is never uniform and the introduction of RDTs has brought with it unintended effects. In the cases standardization has for instance led to a tricky moral negotiation with patients, or brought with it new uncertainties, as pharmacists are transformed into diagnosticians. Overall, the results suggest that RDTs might function best when they can draw on the medical infrastructure that they were designed to extend in the first place.

## Methods

For the last 9 years, this research has followed RDTs into different spaces from expert meetings, to published policy and research documents and the sites in which in which care for malaria is provided. In particular four qualitative case studies have been conducted in Uganda (2), Tanzania and Sierra Leone. Each case draws on a broad set of ethnographic and interview data. The case studies selected are representative of the trends identified in the broader respective studies.

### Uganda I

The field research is part of an ongoing qualitative doctoral research project in medical sociology at Bayreuth University, Germany. Between 2013 and 2014, 20 in-depth interviews and 3 focus-group discussions have been carried out in Mukono District. The interviews are embedded in participant observation in lower level health facilities, document analysis of health reports, evaluations and statistics requested and discussed at the Ugandan Ministry of Health.

### Tanzania

A series of qualitative studies were conducted in northeast Tanzania between 2004 and 2012, to understand the role of malaria diagnostics in hospitals and health centres, starting with microscopy and subsequently RDTs when they were introduced in a pilot study and then in a cluster randomized controlled trial. The first study involved 2 months of observational and in-depth interview work at eight hospitals in 2004, when 22 clinical staff and 39 laboratory staff were interviewed. From 2006–2008, ethnographic fieldwork took place at two hospitals, with additional interviews and a questionnaire survey carried out at a further 11 hospitals and a clinical officer training college. In 2009–10, after a pilot introduction of RDTs at health centres, with minimal staff training, 19 in-depth interviews were carried out with clinicians and 10 focus groups with 103 participants from surrounding communities. In 2011, 6 months into a three-arm cluster randomized trial in which all health centres received RDTs but some had received additional peer-group workshops for clinicians, and some additionally had leaflets for patients, eight in-depth interviews were carried out with clinicians. In 2012, 18 months after the trial began, and after additional interventions of motivational and feedback SMS texts had been instituted in two trial arms, a further 17 in-depth interviews were conducted with clinicians from across the three arms of the trial.

### Sierra Leone

This qualitative study was conducted in Bo District in Sierra Leone in 2011. The aim of this study was to evaluate the viability of decentralizing malaria testing and treatment at village level through community malaria volunteers (CMVs). The study applied a three-pronged, qualitative and ethnographic approach triangulating (1) 20 qualitative, semi-structured interviews with CMVs, (2) four qualitative, in-depth interviews, and (3) participant observation at six CMV meetings, eight community sensitizations and 4 weeks of participant observation with the CMV supervisors on their duties in the villages.

### Uganda II

This study was conducted in Mukono District district between 2011 and 2012 alongside a complex intervention trial and incorporated both quantitative and qualitative methods. Twenty focus group discussions were held with health workers (8); local residents and drug shop clients (5); and drug shop vendors (7). In total 54 DSVs, 54 local residents and 71 health workers took part. In the knowledge that the retail sector was coming under quite considerable political scrutiny, a discourse analysis of articles published between 2009 and 2012 in the government owned newspaper New Vision was conducted identifying 47 stories as relevant to the current study. Quantitative data was gathered in the form of 504 semi structured interviews with customers held 4 days, and again between 10 and 14 days after visiting a drug shop.

All names of research participants mentioned in this article have been fully anonymized.

## Results and discussion

### RDTs work in practice

#### RDTs as a quick and easy diagnostic technology? Integrating RDTs in health centres’ everyday work in Uganda

Despite its assumed simplicity, the introduction of RDTs into lower level facilities with no laboratory in Uganda has been challenging. Most health workers interviewed emphasized the tremendous change the devices brought to their routine provision of health services. The tests first and foremost increase the complexity of everyday work routines by adding another layer of tasks and requirements. Gathered during an informal talk outside the health centre Nicole, a 21 old nursing assistant, described the complexities of her everyday work like this:

Nicole: “One time I was here alone and had to work on over 60 patients…we had all the testing kits and I had to test them as well. So I had to take their history, and as you see I am writing in almost six books here, then go to the other table for testing, then go to the treatment room to give medicine and then back to this table. So that day I realized that at some point, I was writing in the books wrongly. I would write the diagnosis in the place of the treatment. Another time I forgot to write the patients names on the testing kits when I was testing, I think like three patients, and finally I could not remember whose results they were exactly”.

RU: “So, what did you do?”

Nicole: “I just gave all of them Coartem…because two of them were positive and one was negative. So to be on a safe side I gave all of them Coartem.”

While the story illustrates the difficulties in coping with the lack (and absenteeism) of a professional workforce it also reveals how routine work is affected by the technical, performative and administrative requirements of RDTs. Shifting back and forth between six books (that Nicole struggles with) draws attention to the additional—and often not much loved—paperwork and accounting tasks the tests made necessary. While most health workers found it hard to grasp and accept the relevance and meaning of the time consuming data collection, and much manipulation of data can be observed, this is not what interests us here. Nicole’s story shows the difficulties that health workers encounter as it falls to them to document the regulatory potential of RDTs, which goes beyond individual case management and renders the devices productive on a population level.

But Nicole’s story also hints at other complexities that are folded into the diagnostic process with rapid tests. One timesaving practice observed most often was the collection and performance of several tests at once. Performing tests in a consecutive manner allows the health worker (or his/her colleague if available) to use the time he/she would wait for results to do other tests. After 3–6 tests he/she then makes sure all test shows results properly. Doing this well requires detailed attention to the labelling of the tests and allocation of test results to the respective patient. Another tactical adaptation due to high patient loads is the quick reading of results. While training manuals and the manufacturer recommend to wait at least 15 min before reading results, a common practice was to decipher and interpret results after only 2–5 min. Reading results after a short while saves a great deal of time but this practice also needs to put in contrast with the most extreme version of time-saving, namely to skip performance of tests entirely and voluntarily shift back to clinical diagnosis (as observed in some of the health centres facing high patient loads). These are only some selected examples of the creative work health workers employ to render new requirements and intensified workload compatible with their routine work demands, and show how the use of a novel technology is creatively fitted in with already existing work routines. What the cases presented here have in common is that *simple reading* of test results requires multiple and at times complex adaptation practices in order to incorporate the RDTs to a given context of high workload and low staffing. It also shows that the notion of time inscribed in the tests is not perceived as rapid. In direct comparison with clinical diagnosis/presumptive treatment the temporalities of performing a test together with the paper work and data collection, RDTs appear as a rather slow and time consuming device.

#### Creating modern health workers in Tanzania: moral dilemmas of administering RDTs

The series of studies on the role of malaria diagnostics in northeast Tanzania each showed how central the provision of malaria medication was to being able to provide care in a setting in which means of making and managing other diagnoses were largely lacking. Technicians working in under-resourced laboratories were frequently observed to choose to send back positive results for malaria in order to satisfy expectations for malaria. Likewise, clinicians consulted at these health facilities frequently gave a diagnosis of malaria—an expected, acceptable and easily manageable diagnosis.

Pat (Hospital Clinical Officer, 2004): “All cases of fever in adults are usually malaria. If the slide is negative but there are malarial symptoms you should always treat with antimalarials but not antibiotics as there are not many other causes of the same symptoms.”

Through the ethnographic fieldwork in the hospitals it became clear that a diagnosis of ‘malaria’ incorporated a range of ailments which were less easy to identify and treat for both logistical and social reasons, as explained by this clinical officer in 2006:

Oliver: “There are other things that are now arising, with this new HIV/AIDS … Even clinicians don’t think symptoms are AIDS related: they just think malaria, pneumonia and diarrhoeal disease, rather than the HIV. But if they were to think, there would be many diagnoses.”

CC: “[…]Why do clinicians miss HIV?”

Oliver: “You know according to our rules, they can’t screen without counselling, so they have no authority to test without patient consent. So even if they think *“I should think about HIV for this patient”* they cannot. If we could screen like we do with malaria it could be easier, but the policy is not yet. It is the stigma of the disease still. And the counsellors are very few and are busy … So they simply file that we are dealing with malaria or pneumonia.”

Clinicians also explained how they were unable to deal with conditions such as stress, often presented by women, for which anti-malarials were again seen as the best management. Clinicians often spoke of their decisions in terms of moral obligations: to not miss malaria, but also to provide a diagnosis that could be accepted and managed by the patient and their family. These moral obligations continued to be felt, and to create dilemmas as RDTs took hold.

In 2006, RDTs were first introduced into this context of frequent and diverse use of antimalarials. This first trial found anti-malarial prescribing to be entirely unaffected by the use of these new tests [11]. At this time, malaria was publically understood to be the chief cause of fevers, in part due to the public health emphasis on the disease through numerous campaigns and programmes. Meanwhile, epidemiological research was revealing a much reduced incidence of malaria in the area [12]. In a second wider-scale pilot of RDTs in health centres in 2008–9, the high frequency of negative RDTs was still unexpected amongst clinicians and patients, and the tests were therefore considered of dubious quality. In many cases, the tests remained unused and negative results ignored.

In the third trial in the same area, ‘Targeting ACT (TACT)’, the research team set out to investigate what it would take to attain high uptake of testing and adherence to test results [13]. In 2011, clinicians in two of the three arms of the trial received additional interventions beyond training on the use of the test. First, they attended peer-group workshops which sought to underscore epidemiological changes in malaria, and to embed the use of the test in a process of re-fashioning the clinician from ‘old school’ to ‘modern’, with visible government endorsement in content and materials. The training encouraged techniques of self-experimentation and self-observation and health workers often described their pleasure in being able to identify malaria through the test and being able to resist using malaria medication for themselves and their patients when it showed negative. Later, SMS texts were sent to clinicians’ mobile phones with individualized feedback on practice and generic motivational messages, demonstrating a system of monitoring. In this situation, maintaining their new identity as a modern clinician who could adhere to the test result simultaneously created a new type of patient in the health centre, one to be denied medication by health workers staying strong in the face of demands for care. Some health workers described with unease the way that adhering to the test undermined the ‘humanity’ of their relationships with patients, by refusing the only care available to sick patients. They had to convince patients to unlearn or not to trust their bodily perception and disease experience. This caused moral dilemmas that for some clinicians triggered creative action, such as ‘lying’ that adherence to test results was a matter of the law. The RDT thereby emerged not as a tool per guidelines, to enable the treatment of other diseases previously labelled malaria (perhaps unsurprising as no additional diagnostics or medication were provided to enable that to be the case), but rather appeared as a tool of governance, aligning clinician activities with the aims of the state and enabling the reorientation of relationships between health workers, patients and medicines in these previously hard to reach settings in the peripheries of the health system [14].

#### Are RDTs easy to use for everyone? Evidence from community health volunteers in Sierra Leone

Following the implementation of RDTs in rural Sierra Leone made us aware of another challenge in the introduction of RDTs. RDTs as a diagnostic technology are assumed to be so simple in usage that they can be successfully performed by minimally trained personnel, for instance by medically-untrained community health volunteers. In the case study from Sierra Leone it however becomes clear that the simplicity of the RDT is by no means self-evident. Its correct use is rather an achievement. An achievement that requires close supervision and regular training by formally trained health care staff. The mobility of tests then also appears as a mobility that is enabled by a second-tier mobility: the movement of project supervisors to provide remotely living community health workers with supplies and constant supervision.

The project comprised of 50 medically untrained volunteers- community malaria volunteers (CMVs), who diagnosed members of their home communities with rapid diagnostic tests for malaria and administer treatment. At the beginning all CMVs received the 3 days initial training, which had been 3 years ago at the time of the study. However, over the course of the project this training proved to be insufficient. As a result, after 2 years of operation, the number of medically trained supervisors was increased. As one of the supervisors has put it: “Close supervision and regular visits in the village are essential because the CMVs tasks on the farm and in our project are so different. They are not used to this kind of work, and need our help”. In addition most CMVs only received very limited formal education (and in most cases this education was received many years ago). At the time the study was conducted, monthly refresher trainings at the CMV meetings were introduced, which identified several significant challenges that many CMVs struggled with in their performance of RDTs.

Firstly, despite owning watches a significant number of CMVs had problems calculating the time when the RDT was ready to read. Many CHWs had very limited schooling, and did not use mathematical skills in their daily routines; thus adding 15 min waiting time from a given time of day was a difficult task for most volunteers. The challenge was to add 15 min to the time when the test was taken (e.g. 11:52 + 0:15 = 12:07). Very few CMVs were able to calculate correctly in a mental calculation, some needed to do a written calculation, and some were only able to achieve the correct result by using their fingers to count. The count calculations were improved through the training at monthly meetings, but need to continue regularly in order for all CMVs to do reliable calculations. Furthermore, the concept of 24-hour clock is not used in most communities’ regular lives. For instance, in one community sensitization the community assumed that 1 day is 12 h rather than 24. This would suggest to use locally used measures of time (i.e. 1 day rather than 24 h) in the medical protocols.

Secondly, most CMVs struggled with the safe application of RDTs. CMVs did not know what the dangers of unsterile needles were, equated ‘sterile’ with ‘clean’, and showed a rather careless handling of needles (not using gloves, not disposing the needle immediately, putting the needle cap on again after needle use and then reusing needles, not using the safe container for disposal). Thirdly, a majority of CMVs had difficulties in recalling (all) signs of simple and severe malaria, or the correct treatment regimes. Fourthly, minor mistakes in record keeping were frequent. If CMVs made mistakes in the recording it was mostly due to the fact that the need for this specific record, its purpose and the further processing of the records by the data management team were not understood fully. These results points not only to a need for more regular and systematic training, but also show that emphasis during the trainings needs to be put on *why* certain procedures need to be followed in order for these to become a routine practice for the CMVs. In short, while RDTs are perceived to be simple to use correctly and safely, this case study shows that is not self-evident. If RDTs are simple or difficult is rather dependent on the context of its use, in this case on the education, training and daily routines of the community volunteers.

#### Creating legitimacy—RDTs as a shortcut to expertise for drug shop vendors in Uganda

In research conducted in Ugandan drug shops in the largely urban district of Mukono, RDTs also extended the amount of time that drug shop vendors took to see clients. The drug shops in the study were varied in terms of their structures and the services that they offered. Some worked more as small clinics with separate rooms in which in-patient services were provided; while others appeared much more as small shops in which medicines were likely to be purchased on demand. DSVs described how some of these shops were ill equipped to provide space and chairs for patients waiting either to be tested or for test results. Moreover, one of the key attractions of those typically seeking care in these spaces was considered to be the quick services available in the shop (often contrasted with the long waiting times at health centres). Some DSVs raised concerns about the introduction of the test, that demands on time risked the loss of clients who wanted to purchase of medicine without either waiting to be tested or waiting if the DSV was attending to others.

Yet, as the test was introduced by the trial it was accompanied by other quite substantial shifts in care-giving in these spaces. Rather than raising concerns about the increases in time spent in the shop and on each client, many clients and DSVs described the trial and the introduction of blood based diagnostics as a transformation in the services and expertise on offer. The act of undertaking the test in the shop was largely welcomed (by both DSVs and clients). Clients appreciated that they no longer had to attend larger health facilities to gain access to knowledge that a blood test would provide when they were unwell. They often requested further increases in services, and larger waiting rooms. The DSVs described it as a success, because it was leading to an increase in clients, making their business more profitable. For many DSVs, the introduction of RDTs into the shops was interpreted as the beginning of a trajectory towards closer associations with larger health centres, and they hoped that this would lead to the creation of a professional identity for them. Testing as a performance at the front of the shop marked the DSV out as the equivalent of a professional health worker, an expert usually only found in large health facilities. For both clients and DSVs testing rendered DSVs into a medical expert able to take blood, use technology and the test results to provide (more) effective medication [15]. The tests were embedded in a discourse of change that revolved around a shift in the DSV away from previous, less effective practices of guesswork that matched a patients’ symptoms to an appropriate medication to being able to know disease (not just malaria) and thus to know what it was that they were treating. Yet, in their implementation RDTs have the potential to create a group of patients without a diagnosis, namely if a patients tests negative for malaria. In the shops we studied this uncertainty was managed through increasing sales of medication. Patients in the control arm of the trial (in drug shops where no RDTs were present) spent least on medication, followed by those testing positive with RDTs, and with those testing negative making on average the highest payments for medication. The RDT, billed at global policy level as so easy to use that those with almost no medical training to can carry it out, emerged in the retail sector as enabling expertise and professionalism to come into being in the person conducting the test. However, as has been shown, business and medical interests of the DSVs mix, and the expertise coming with testing results in ambivalence. On the one hand can patients save time and get a parasitological diagnosis quicker than in health centres, on the other hand if patients test negative, they (and the DSV) revert back to an earlier form of guesswork-expertise, resulting in higher drug sales. For the business of the DSVs a good result, however for the health of the DSVs’ clients the results are less clear-cut.

## Discussion

These four case studies illustrate that making the presumed simple and mobile technology of RDTs work in practice is not as straightforward as tends to be assumed in the public health discourse around RDTs. This study suggests that taking the issues reported on seriously goes beyond iterating a need for more regular and systematic training. The first two case studies show how much work is required to incorporate RDTs into regular clinical practice. For clinicians, this means reworking established organizational practice within facilities and communities. It requires substantial investment in the logistics of record and time keeping, the management of the—often perceived as rather fiddly—RDT kit, and in finding organizational and physical space for testing. Crucially, this shifting of organizational practice includes reworking obligations towards the priorities of the state, which brings with it moral dilemmas in clinicians’ interactions with patients. This is particularly true for patients who test negative in settings where no further diagnostic and treatment capacities are at hand. Often then, the clinicians spoken to are confronted with the moral dilemma of either adhering to the test and issuing referrals that they know are often not adhered to, or prescribing antimalarials despite a negative test. These case studies show that while the tests may be simple to use technically, they are not simple in terms of their organizational demands and with regards to the moral choices involved in navigating good care for patients.

The second set of case studies questions the suitability of RDTs for DSVs and CMVs more fundamentally. They indicate a need for critical awareness that any assumption of what is ‘simple’ is highly context-dependent and certainly not universal. For people with very limited formal education the safe and effective application of RDTs might indeed not be straight-forward, which questions the assumption of RDTs being able to empower communities cut-off from medical infrastructure. The framing of RDTs as universally easy to use and requiring lower training requirements is misleading in the sense that it compares RDTs to microscopy. Indeed, RDTs need lower level of skills than microscopy or other diagnostic technologies, but the question is for whom. As has been shown the users are not the same either: they are not skilled laboratory technicians, but often unskilled in medical and/or laboratory work. Thus, the training requirements are different and cannot simply be described as ‘lower’. Similarly, taking the case studies of DSVs and CMVs together shows that expertise is not something that technology can simply substitute or enact, but a learned skill, an achievement. The results suggest that these problems encountered are not behavioural in the sense that they concern (in)competent individuals, but they are rather structural issues pertaining to the use of RDTs by low-skilled workers. These case studies point to the limits of circumventing medical expertise and infrastructures with technology. They do not discredit the technology itself, but rather point to the complex relations between a technology, its specific users and health systems—as well as their capabilities and constraints.

The case studies question the simplicity of RDTs, and their potential to standardize diagnostic procedures and enable clear parasitological diagnosis for everyone. In all case studies RDTs do not as such standardize the diagnosis of malaria in the sense that diagnosis becomes definite and clear-cut. The process of standardization in practice is rather characterized as a process of negotiation between health workers and patients. Furthermore, as the results show diagnosis is not merely a technological procedure, but involves moral questions based on health workers’ perceptions of the patient’s alternatives in terms of diagnosis and treatment as well as alignment with broader political economic agendas. Standardized (and correct) diagnosis depends on the time available to the health worker in clinical practice, and on DSVs and CMVs ability to perform the test correctly. Furthermore, for patients being tested by DSVs a negative malaria test has not led patients to receive a better diagnosis or evidence-based care, but has rather resulted in the patients being subjected to more ‘trial and error’ drug sales. For DSVs performing a RDT is first and foremost part of their commercial enterprise and not of clinical practice. These logics, however, are intermingled in a problematic manner: DSVs appreciate tests because they increase their authority, which in turn helps their business. Patients appreciate the time-saved when consulting a DSV. What is less clear, however, is if improved diagnostics at drug stores lead to better treatment.

Social scientists have drawn attention to the multiplicity of diseases (for example, [16]) which can be seen to have different functions, roles, significance in different spaces and for different actors who are enacting the disease. This study suggests that malaria diagnosis too is manifest differently in different places and from different perspectives. Nevertheless substantial funding goes into standardized technological solutions such as RDTs. A key aspiration for these technologies is to make them as universal as possible—so usable across many different places and by different people. As has been shown however, many of the challenges in their implementation revolve around conflicts between local needs and these universal solutions. This study suggests that rather than being surprised when such technologies are misappropriated or rejected in different places, one should be surprised at the assumption embedded in such technological solutions—that the problem is universal and can be dealt with through universal solutions. In the implementation of RDTs in different settings one can see that simple technologies too come with many complexities, which nuance the assumptions of simplicity and their capacity to standardize malaria diagnosis across a variety of settings. To repeat, this is not a problem with the technology itself, but indicative of a problem in the relation between technology, users and health systems. Thus, this study advocates for more careful and systematic attention in the global health community to processes of local translation and appropriation of technology.

When health care workers, DSVs and CMVs performed RDTs, negotiated results and their consequences with their patients not only were moral issues at stake, but many more politically contentious processes were underway. The focus of public health interventions on health worker ‘behaviour’ and technological solutions reflects the tendency to render the causes of ill health technical and intervenable in ways that can be counted, evaluated and targeted. However, confronting the complexities of RDTs with more education and governing health worker behaviour stops short of a critical appraisal of RDT capacities as a diagnostic, clinical and social technology. RDTs in the case studies presented have emerged as technology that is seductively simple to use as a stand-alone tool, but not quite as simple to make to work effectively in a variety of high-pressured health care settings. Health care settings that are characterized by high patient loads, understaffed and underpaid personnel, and under-equipped in terms of general and specific diagnostic and treatment capacities. Indeed, it has been showed that to perform RDTs effectively might very well need exactly the infrastructure they were designed to substitute: the medical expertise, organizational capacity and diagnostic and treatment options of functioning health systems. These results do not suggest to forgo RDTs in the future, but they do underline that successful malaria diagnosis and treatment requires at least as much investments in general health infrastructure as it does in new technologies.
